# UWF-SS-OCTA evaluation of changes of choroidal microvasculature and microstructure in adult patients with high myopia and relationship with age and axial length

**DOI:** 10.3389/fmed.2026.1784480

**Published:** 2026-04-24

**Authors:** Rong Di, Haocheng Nan, Fei Li, Jiameng Zhang, Wanni Zou

**Affiliations:** 1 The First Affiliated Hospital of Xi’an Jiaotong University, Xi’an, Shaanxi, China; 2 Shaanxi Provincial People’s Hospital, Xi’an, Shaanxi, China; 3 Ankang People’s Hospital, Ankang, Shaanxi, China; 4 Xi’an People’s Hospital, Xi’an, Shaanxi, China; 5 Xi’an Jiaotong University Health Science Center, Xi’an, Shaanxi, China

**Keywords:** axial length, choroid, choroidal capillaries, high myopia, swept-source optical coherence tomography angiography

## Abstract

**Objective:**

This study aimed to evaluate the changes of choroidal microvasculature and microstructure in adult patients with high myopia by ultra-wide-field swept-source optical coherence tomography angiography (UWF-SS-OCTA) and explore the relationship with age and axial length.

**Methods:**

Adopting a cross-sectional study design, 15 normal subjects (26 eyes) admitted from January 2025 to June 2025 were enrolled as control group, and 39 patients with high myopia (72 eyes) were collected as high myopia group. Each eye underwent 6 mm × 6 mm and 24 mm × 20 mm SS-OCTA scans centered on the macular central fovea and optic disk, respectively. Each B-scan position was scanned four times, with the mean value taken. Macular thickness, axial length (AL), spherical equivalent (SE), linear density, choriocapillaris flow density (CCD), three-dimensional choroidal vascularity index (3D-CVI) and choroidal vascular volume per unit area (CVV/A) were compared between high myopia group and control group. Multivariate linear regression analysis was conducted to analyze the relationship of UWF-SS-OCTA indicators with AL and SE.

**Results:**

We calculated the average value for all the regions. No statistical differences in mean linear density, CCD, 3D-CVI and CVV/A under 6 mm × 6 mm scan were exhibited between control group and high myopia group (*p* = 0.176, 0.415, 0.358, 0.177). Patients with high myopia showed reduced mean CCD, 3D-CVI and CVV/A under 24 mm × 20 mm scan compared to control group (*p* = 0.003, 0.026, 0.005). Spearman correlation coefficient for correlation analysis suggested that the mean linear density, CCD, 3D-CVI and CVV/A were negatively correlated with AL in high myopia group (*r* = −0.51, −0.33, −0.53, −0.44, *p* < 0.001), and were positively associated with SE (*r* = 0.33, 0.26, 0.28, 0.37, *p* < 0.05). Taking AL as the dependent variable and incorporating age, eye side, mean linear density, CCD, 3D-CVI, CVV/A and SE as independent variables, multivariate linear regression analysis found that CCD (β: −0.25; 95% CI: −0.44 ∼−0.07) and SE (β: −0.16; 95% CI: −0.24 ∼−0.08) were independently associated with AL.

**Conclusion:**

Patients with high myopia exhibit reduced mean CCD, 3D-CVI and CVV/A. Higher mean CCD value correlates with shorter axial length.

## 1 Introduction

Myopia is an important cause of correctable visual impairment, even among young population in the world. It is estimated that by 2050, 5 billion and 1 billion individuals worldwide will be affected by myopia and high myopia, respectively (1). High myopia is generally defined as spherical equivalent (SE) < −6.00 D or axial length (AL) ≥ 26.00 or 26.50 mm, which can be divided into simple high myopia and pathological high myopia. Simple high myopia can lead to myopia-related macular lesions, thus evolving into pathological high myopia (PM) (2). The increasing prevalence rate of high myopia, particularly in the Asian population, highlights the importance of taking action to prevent the progression of high myopia from causing ocular complications and resulting in visual loss (3–5). However, the progression of high myopia is significantly associated with changes of retinal choroidal blood flow and thickness (6, 7). Therefore, the changes of blood perfusion and thickness of retinal choroid in high myopia have been a critical research topic for decades, and also one of the focuses of ophthalmologists in clinical practice. Quantitative analysis of retinal choroid in high myopia may provide vital clues for understanding the pathophysiological characteristics of high myopia-related diseases and clinical diagnosis of high myopia.

Abnormal choroidal blood volume and/or impaired blood flow can cause photoreceptor dysfunction and death. Currently, it is believed that the changes of choroidal vascular structure and blood flow may play an important role in the progression of myopic fundus lesions. Some studies have pointed out that the choroid tends to become thinner when the retinal thickness remains stable in the early stage of myopia. In the observation of high myopia, it is also found that choroidal tissue will undergo abnormal changes before retinopathy. Ho et al. (8) have explicitly stated that decreased visual acuity in myopic patients is only linked with the change of choroid at the macular central fovea. Recently, Wu et al. (9) creatively put forward a new hypothesis on the pathogenesis of myopia: external visual stimulation may change hemodynamics and hemorheology and result in hypoxia in scleral microenvironment by regulating choroidal blood circulation, thereby triggering the occurrence of myopia. The proposal of this theory provides new ideas and directions for the role mechanism of choroid in the pathogenesis of myopia.

In recent years, with the advancement of optical coherence tomography angiography, attention has been paid to the medium and large vascular layers of the choroid. However, most studies have focused on two-dimensional quantitative analysis of choroidal lumen and stroma on a single OCT B-scan image obtained by scanning the macular central foveal region (10–12). As a non-invasive vascular imaging technique, swept-souree optical coherence tomography angiography (SS-OCTA) possesses faster scanning speed, longer wavelength and deeper penetration ability, making it possible to quantify the blood perfusion changes of the medium and large vascular layers of the choroid (13). Recently, the built-in deep learning-based software of SS-OCTA instrument enables the quantitative analysis of three-dimensional blood flow and thickness changes of choroid in high myopia, which is a major progress different from two-dimensional quantitative analysis. Furthermore, its good repeatability can provide more comprehensive and accurate information on the changes of retinal choroidal blood flow and thickness (14). Although significant progress has been made in the related fields, the specific role mechanism of choroidal microvasculature and microstructure in the progression of myopia has not yet been fully clarified. The relationship between changes of choroidal layers and progression of myopia as well as the relationship between choroidal blood flow changes and fundus pathological changes in high myopia remain unclear. Therefore, we adopt ultra-wide-field swept-source optical coherence tomography angiography (UWF-SS-OCTA) with scanning ranges of 6 mm × 6 mm and 24 mm × 20 mm to quantitatively compare the choroidal vascular changes in high myopia eyes and normal control eyes, with the aim of deepening the understanding of pathophysiological mechanism of fundus changes in high myopia, and providing a reference for the prevention and control of high myopia and its fundus pathological changes.

## 2 Materials and methods

### 2.1 Study design

This cross-sectional study included 15 normal subjects (26 eyes) who received 6 mm × 6 mm and 24 mm × 20 mm SS-OCTA scans between January 2025 and June 2025 as control group and 39 patients with high myopia (72 eyes) as high myopia group. The control group was matched by gender and age (± 3 years). The research was approved by the hospital’s medical ethics committee and adhered to the Declaration of the Helsinki. All subjects provided written informed consent.

### 2.2 Study population

Inclusion criteria for control group: (1) Age range: 18–65 years old; (2) Best-corrected visual acuity > 0.8; (3) SE ranging from −3.0 to +1.0 D; (4) Intraocular pressure between 10 and 21 mmHg (1 mmHg = 0.133 kPa). Inclusion criteria for high myopia group: (1) Age ≥ 18 years old; (2) SE < −6.0 D or AL > 26.5 mm; (3) Intraocular pressure ranging from 10 to 21 mmHg. Exclusion criteria: (1) History of systemic diseases such as hypertension and diabetes mellitus; (2) History of vitreous surgery or intravitreal injection therapy; (3) History of fundus diseases including diabetic retinopathy and retinal detachment; (4) Inability to obtain high-quality fundus images due to poor eye fixation or severe opacity of dioptric media; (5) Voluntary withdrawal from the study; (6) Concurrent systemic diseases affecting the fundus, such as Marfan syndrome and ankylosing spondylitis; (7) Image signal intensity below 8 and presence of substantial motion artifacts or central shift.

### 2.3 Methods

#### 2.3.1 Conventional examination

SE was determined by fully automatic refractometer, and intraocular pressure was measured by non-contact tonometer, and AL was detected by IOLMaster intraocular lens biometer, with five measurements averaged. Compound tropicamide eye drops were administered for pupillary dilation, followed by fundus examination with pre-set lens to rule out other organic ocular diseases. All examination items and data collection were completed by the same clinician.

#### 2.3.2 SS-OCTA examination

The instrument used was SS-OCTA (SVision Imaging, Henan, China), with the SS laser set to a central wavelength of approximately 1,050 nm and a scanning speed of 200,000 A-scans per second. The axial resolution, lateral resolution and scanning depth were 5 μm, 15 μm and 3 mm, respectively. Each subject received the examination in a dark room between 2:00 PM and 5:00 PM. A 10-min meditation period preceded the examination to minimize the influence of diurnal variations in choroidal blood flow perfusion and other confounding factors.

Each eye underwent 6 mm × 6 mm and 24 mm × 20 mm SS-OCTA scans centered on the macular central fovea and optic disk, respectively. Each B-scan position was scanned four times, with the mean value taken. To eliminate the influence of ocular motion artifacts, the system incorporated an eye-tracking tool based on an integrated confocal scanning laser ophthalmoscope. A trained examiner scanned the same region of each eye twice, and then selected the higher-quality image from the two generated scanning images for subsequent quantitative analysis.

Using Littmann’s method and Bennett’s formula, the image size was adjusted according to the AL of each eye. The larger the AL, the larger the obtained image. In this study, AL correction was performed for each image based on the correction coefficients. After automatic segmentation using the proprietary built-in algorithm in the SS-OCTA device, trained technicians (HL) checked and manually modified the incorrect segmentations. Subsequently, the deep learning algorithm in the SS-OCTA device automatically quantified the three-dimensional choroidal vascular index (3D-CVI) of the entire scanning area, choroidal capillary blood flow density (CCD), and choroidal vascular volume per unit area (CVV/A). 3D-CVI is the ratio of the choroidal vascular lumen volume to the choroidal volume of the selected area × 100%, and CCD is the ratio of the choroidal capillary blood flow signal area to the selected area × 100%. 6 mm × 6 mm scanning range included the macular, 3 cm superior, 6 cm superior, 3 cm inferior, 6 cm inferior, 3 cm temporal, 6 cm temporal, 3 cm nasal and 6 cm nasal regions. 24 mm × 20 mm scanning range comprised the superior temporal, superior, superior nasal, temporal, macular, nasal, inferior temporal, inferior and inferior nasal regions.

**FIGURE 1:**
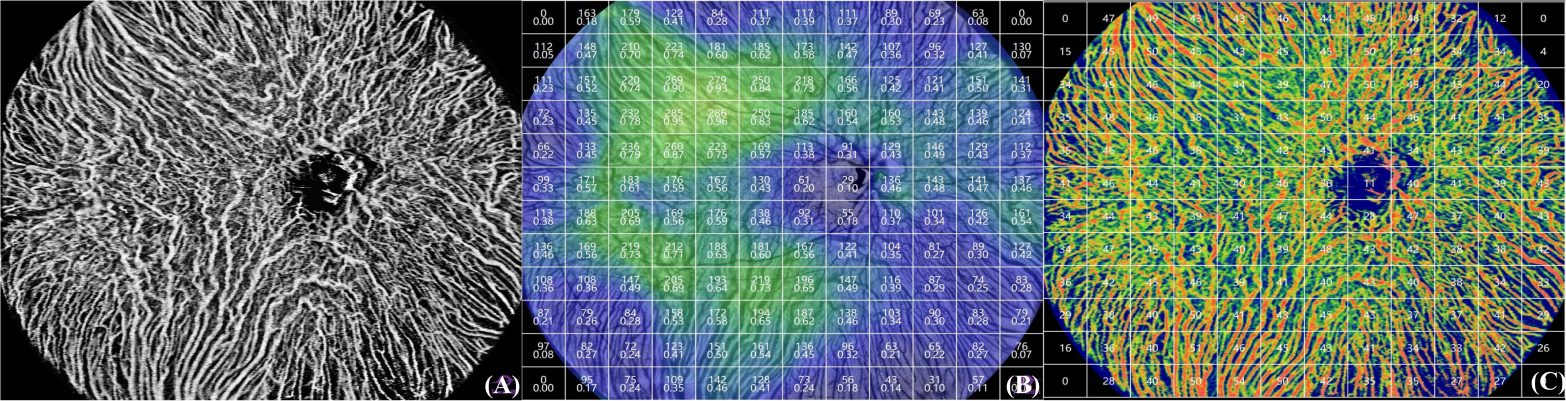
A classic case. **(A)** Choroidal blood flow structure of 1 patient with high myopia under 24 mm × 20 mm scan; **(B)** CT; **(C)** CVI.

The repeatability of SS-OCTA measurements was determined using intraclass correlation coefficients (ICCs) and repeatability coefficients. Ten healthy individuals’ both eyes were scanned twice by an experienced observer, and then re-scanned by the same observer 3 days later. The repeatability coefficient of the measurement results was 1.72 times the standard deviation of the changes in the two measurement values, indicating that the OCTA measurement values have high reproducibility. UWF-SS-OCTA analysis diagram of a classic case was displayed in Figure 1.

### 2.4 Ethical principles

(1) Principle of voluntariness: All patients provided informed consent and could withdraw at any time during the study.
(2) Principle of confidentiality: All data during the study process were kept by the team, and were not allowed to be used for any commercial purposes except for article publication and ethical review. The data recorded and used during the study process were encoded to ensure no infringement of personal privacy of patients.
(3) Principle of safety: Regardless of the grouping conditions, the study process would not cause any potential harm to the study subjects, nor would it affect their original treatment regimens and outcomes.

### 2.5 Statistical analysis

SPSS 27.0 software (SPSS Inc., Chicago, IL, USA) was adopted to analyze the above data. Continuous data consistent with normal distribution were represented as Mean ± SD, and differences between two groups were compared by independent-samples *t*-test. Continuous data conforming to skewed distribution were denoted as [M (Q_1_, Q_3_)], and Mann–Whitney U test was applied for between-group comparison. Categorical data were expressed as *n* (%) by adopting chi-square test or Fisher’s exact test. Significant level was considered as α = 0.05.

## 3 Results

### 3.1 Demographic characteristics

15 normal subjects (26 eyes) were included in control group, and 39 patients with high myopia (72 eyes) were classified as high myopia group. As shown in Table 1, the differences in AL (*p* < 0.001) and SE (*p* < 0.001) between the two groups were statistically significant, and the age (*p* = 0.291), mean central thickness (*p* = 0.063), intraocular pressure (*p* = 0.091) and eye side (*p* = 0.581) exhibited no statistical differences.

**TABLE 1:** Demographic characteristics.

Clinical data	Control group (26 eyes)	High myopia group (72 eyes)	Statistic	*P*
Age	41.33 ± 8.16	38.97 ± 6.93	*t* = −1.06	0.291
Mean central thickness, M (Q_1_, Q_3_)	282.00 (272.00, 300.00)	275.00 (262.75, 289.00)	Z = −1.86	0.063
AL, M (Q_1_, Q_3_)	23.70 (22.72, 24.09)	26.75 (26.12, 27.24)	Z = −7.50	< 0.001
Intraocular pressure, M (Q_1_, Q_3_)	14.35 (12.98, 15.28)	13.30 (11.50, 14.93)	Z = −1.69	0.091
SE, M (Q_1_, Q_3_)	−0.50 (−0.94, 0.25)	−9.00 (−10.06, −7.50)	Z = −7.27	< 0.001
Eye side, *n* (%)			χ^2^ = 0.30	0.581
OD	11 (42.31)	35 (48.61)		
OS	15 (57.69)	37 (51.39)		

M, median; Q_1_, 1st quartile; Q_3_, 3rd quartile; Z, Mann–Whitney test.

### 3.2 Comparison of CCD, 3D-CVI and CVV/A between high myopia group and control group

To control for the correlation between the two eyes of the same subject, we reanalyzed the key indicators using the Generalized Estimating Equation (GEE) and used the eye side as the clustering variable. The interaction effect of the 6 mm × 6 mm and 24 mm × 20 mm linear densities was not significant (Wald χ^2^ = 3.14, 4.02, *P* = 0.076, 0.071). The interaction effect of the 6 mm × 6 mm and 24 mm × 20 mm CCD was not significant (Wald χ^2^ = 2.28, 2.72, *P* = 0.118, 0.101). The interaction effect of the 6 mm × 6 mm and 24 mm × 20 mm 3D-CVI was significant (Wald χ^2^ = 9.82, 10.14, *P* < 0.001). The interaction effect of the 6 mm × 6 mm and 24 mm × 20 mm CCD was significant (Wald χ^2^ = 12.34, 10.12, *P* < 0.001).

We calculated the average value for all the regions. As indicated in Table 2, there were no statistical differences in mean linear density, CCD, 3D-CVI and CVV/A under 6 mm × 6 mm scan between control group and high myopia group (*p* = 0.176, 0.415, 0.358, 0.177). Patients with high myopia showed reduced mean CCD, 3D-CVI and CVV/A under 24 mm × 20 mm scan compared to control group (*p* = 0.003, 0.026, 0.005).

**TABLE 2:** Comparison of CCD, 3D-CVI and CVV/A between high myopia group and control group.

Data type	Range	Control group (26 eyes)	High myopia group (72 eyes)	Statistic	*P*
Linear density	6 mm × 6 mm	14.13 (11.05, 16.28)	15.35 (12.10, 17.16)	Z = 1.38	0.176
	24 mm × 20 mm	6.69 (6.41, 7.34)	6.71 (5.95, 7.36)	Z = 0.54	0.590
CCD	6 mm × 6 mm	49.20 (48.00, 50.25)	49.50 (46.00, 52.00)	Z = 0.83	0.415
	24 mm × 20 mm	46.00 (45.00, 47.25)	48.00 (46.00, 48.00)	Z = 3.00	0.003
3D-CVI	6 mm × 6 mm	43.00 (38.25, 43.75)	42.00 (38.50, 44.25)	Z = 0.92	0.358
	24 mm × 20 mm	41.00 (40.00, 42.75)	43.00 (40.75, 44.00)	Z = 2.23	0.026
CVV/A	6 mm × 6 mm	14.50 (11.50, 16.00)	15.50 (12.25, 17.25)	Z = 1.36	0.177
	24 mm × 20 mm	78.00 (64.75, 89.25)	67.50 (52.50, 81.00)	Z = 2.83	0.005

### 3.3 Correlation of mean linear density, CCD, 3D-CVI and CVV/A with AL and SE in high myopia group under 24 mm × 20 mm scan

**FIGURE 2:**
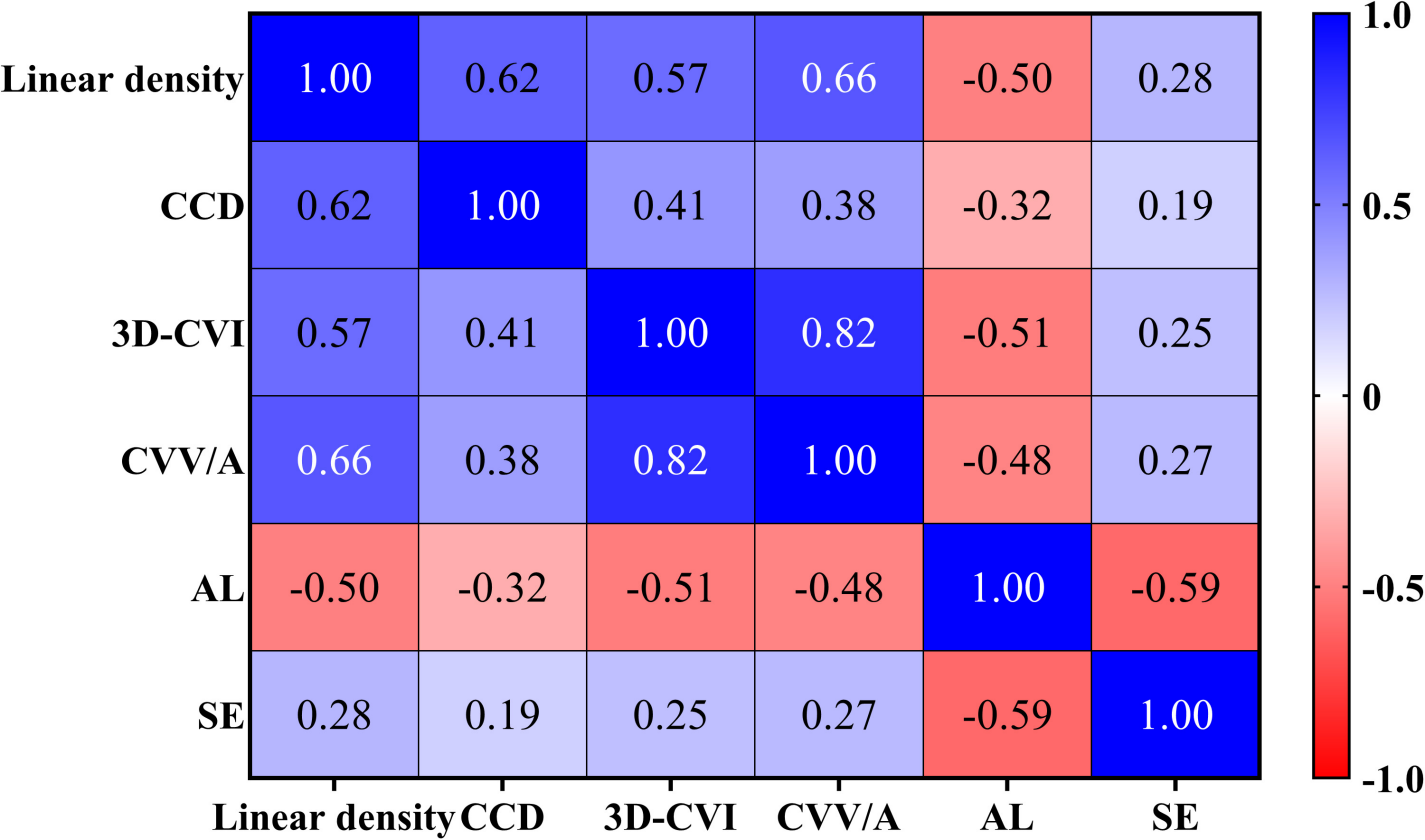
Correlation heat map.

The mean linear density, CCD, 3D-CVI and CVV/A in high myopia group were calculated under 24 mm × 20 mm scan, and descriptive statistics were shown in Table 3. After eliminating confounding factors (age and eye side), we used Spearman correlation analysis and partial correlation analysis to analyze the correlations in this study. As detailed in Figure 2, the mean linear density, CCD, 3D-CVI and CVV/A in high myopia group exhibited negative correlations with AL (*r* = −0.51, −0.33, −0.53, −0.44, *p* < 0.001), and showed positive correlations with SE (*r* = 0.33, 0.26, 0.28, 0.37, *p* < 0.05).

**TABLE 3:** Descriptive statistics of mean CCD, 3D-CVI and CVV/A.

Statistic	Linear density	CCD	3D-CVI	CVV/A
*N*	72.00	72.00	72.00	72.00
Mean	6.63	47.04	42.07	65.81
SD	0.94	1.90	3.08	19.55
Min	4.39	41.00	31.00	25.00
Max	8.39	50.00	48.00	117.00
Median	6.71	48.00	43.00	67.50
P25	5.95	46.00	40.75	52.50
P75	7.36	48.00	44.00	81.00
IQR	1.41	2.00	3.25	28.50
SE	0.11	0.22	0.36	2.30
95% CI	6.41–6.85	46.60–47.49	41.35–42.79	61.21–70.40

**FIGURE 3:**
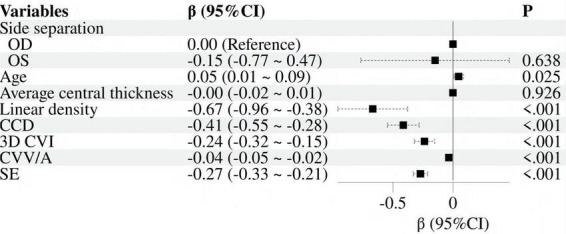
Forest plot of univariate linear regression.

**FIGURE 4:**
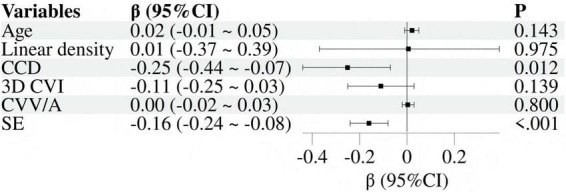
Forest plot of multivariate linear regression.

Took AL as the dependent variable and incorporated age, eye side, mean linear density, CCD, 3D-CVI, CVV/A and SE as independent variables. Before regression, calculate the variance inflation factor (VIF). A VIF value greater than 5 indicates moderate collinearity, a value greater than 10 indicates severe collinearity, and a value greater than 10 indicates extremely severe collinearity. Variable selection is based on VIF being less than 5. After testing, the VIF values of the above indicators are, respectively, 1.124, 1.294, 1.127, 1.092, 1.184, 1.210, and 1.109, indicating that there was no significant collinearity among these variables. The results of univariate linear regression analysis were displayed in Figure 3. The data with *p* < 0.05 were screened for further multivariate linear regression analysis, with results presented in Figure 4. It was found that CCD (β: −0.25; 95% CI: −0.44 ∼−0.07) and SE (β: −0.16; 95% CI: −0.24 ∼−0.08) revealed independent associations with AL.

## 4 Discussion

Both choroidal vessels and stromal components may be affected by different ocular diseases, but choroidal thickness changes cannot clearly indicate whether the alteration stems from blood flow or stromal components. CVI is measured by examining the ratio of the choroidal vascular lumen area to the total choroidal area, and higher CVI indicates greater percentage of vessels in each region of the choroid (15). In the normal population, CVI is not influenced by physiological factors such as age, AL and intraocular pressure, and may be related to choroidal thickness.

Scherm et al. (16) have found that choroidal capillary blood flow perfusion is maintained at a relatively stable level despite increasing physiological myopia. Milani et al. (17) have also reported no statistically significant difference in choroidal capillary layer perfusion area between myopia group and control group. Further investigation by Al-Sheikh et al. (18) on patients with high myopia has revealed increases in both the mean and total flowing cavity areas of the choroidal capillary layer. Conversely, Mo et al. (19) have discovered reduced choroidal capillary density exclusively within pathological myopia group. The results of this study are similar to those of Li et al. (20) and Xu et al. (21). The study results of Li et al. (20) on children with low-to-moderate myopia have demonstrated a significant negative correlation between choroidal luminal area (LA) and AL. Xu et al. (21) have also pointed out that the choroidal luminal volume (LV) in moderate-to-high myopia group is significantly reduced compared to low myopia group. Collectively, these findings indicate that the choroidal blood flow reduction is associated with the progression of myopia. However, whether myopia causes the changes of choroidal blood flow or whether choroidal blood flow changes trigger myopia remains to be further investigated to determine the causal relationship between the two. In this study, CCD (β: −0.25; 95% CI: −0.44 ∼−0.07) and SE (β: −0.16; 95% CI: −0.24 ∼−0.08) were found to be independently correlated with AL. Additionally, another major finding of this study is that CVV/A gradually decreases with increasing myopia severity. The CVV/A decline indicates that the large and medium choroidal blood vessels gradually become damaged as the eyeball becomes overly elongated, suggesting a possible close relationship between choroidal blood flow and myopia. This study is based on a cross-sectional design, which inherently limits the ability to infer causality or temporal relationships. Therefore, our research results only indicate that the higher the average CCD value, the shorter the eye axis. However, they cannot determine the causal relationship between the two.

3D-CVI is the ratio of choroidal vascular lumen volume to total choroidal volume, representing a novel biometric parameter. Calculating 3D-CVI changes accurately provides the proportions of large and medium choroidal vascular volumes, serving as an imaging marker reflecting choroidal perfusion status (22). OCT-based carrier studies have suggested that CVI of myopic eyes decreases with increasing AL, and exhibits no significant correlation with choroidal thickness (CT). Consistent with prior findings, this study demonstrated a negative correlation between 3D-CVI and AL. However, Agrawal et al. (23) have reached conclusions contrary to this study, finding a significant positive correlation between macular CT and CVI. Their study subjects are older (45–85 years old) and subjects with SE < −6D are excluded. Moreover, blood vessels larger than 100 μm are excluded during image processing. These factors may account for the discrepancy with this study. The currently widely accepted conclusion is that compared to CT, CVI is less susceptible to systemic factors such as age, gender, AL, intraocular pressure, choroidal vascular area and blood pressure. Its coefficient of variation is significantly lower than that of CT, making it a more stable and reliable indicator for assessing choroidal vascular status. However, no independent association was observed between 3D-CVI (β: −0.11; 95% CI: −0.25 ∼ 0.03) and AL in this study, which is considered to be related to insufficient sample size.

This study has several limitations. Firstly, this study is a cross-sectional study lacking long-term follow-up of longitudinal cases. It only includes patients with high myopia and fails to incorporate patients with different degrees of myopia for analysis. The relationship between CVI and CT remains controversial in current study findings, which is influenced by factors such as the age of the included population, diopter, choroidal blood flow measurement method and the use of different examination instruments. Finally, this study fails to consider important confounding factors such as fluctuations in ocular perfusion pressure and blood pressure. In summary, this study finds that patients with high myopia demonstrate reduced mean CCD, 3D-CVI and CVV/A, and higher mean CCD value represents shorter AL value. These findings of this study contribute to elucidating the relationship between choroid and myopia progression, providing a theoretical basis for potential mechanisms of myopia occurrence and progression to a certain extent. Furthermore, 24 mm × 20 mm SS-OCTA enables detailed visualization of choroidal structure and quantification of blood flow status, and may serve as an effective tool for clinical evaluation and prevention of choroidal changes during myopia progression.

## Data Availability

The original contributions presented in this study are included in this article/supplementary material, further inquiries can be directed to the corresponding author.
